# 3D-Printed Graphene Nanoplatelets/Polymer Foams for Low/Medium-Pressure Sensors

**DOI:** 10.3390/s23167054

**Published:** 2023-08-09

**Authors:** Marco Fortunato, Luca Pacitto, Nicola Pesce, Alessio Tamburrano

**Affiliations:** 1 Department of Astronautical, Electrical and Energy Engineering (DIAEE), Sapienza University of Rome, 00184 Rome, Italy; luca.pacitto@gmail.com (L.P.); nicola.pesce@uniroma1.it (N.P.); alessio.tamburrano@uniroma1.it (A.T.); 2 Nanotechnology Research Center Applied to Engineering (CNIS), Sapienza University of Rome, 00185 Rome, Italy

**Keywords:** 3D printing, foam, pressure sensor, graphene, nanoplatelets, piezoresistivity, wearable devices

## Abstract

The increasing interest in wearable devices for health monitoring, illness prevention, and human motion detection has driven research towards developing novel and cost-effective solutions for highly sensitive flexible sensors. The objective of this work is to develop innovative piezoresistive pressure sensors utilizing two types of 3D porous flexible open-cell foams: Grid and triply periodic minimal surface structures. These foams will be produced through a procedure involving the 3D printing of sacrificial templates, followed by infiltration with various low-viscosity polymers, leaching, and ultimately coating the pores with graphene nanoplatelets (GNPs). Additive manufacturing enables precise control over the shape and dimensions of the structure by manipulating geometric parameters during the design phase. This control extends to the piezoresistive response of the sensors, which is achieved by infiltrating the foams with varying concentrations of a colloidal suspension of GNPs. To examine the morphology of the produced materials, field emission scanning electron microscopy (FE-SEM) is employed, while mechanical and piezoresistive behavior are investigated through quasi-static uniaxial compression tests. The results obtained indicate that the optimized grid-based structure sensors, manufactured using the commercial polymer Solaris, exhibit the highest sensitivity compared to other tested samples. These sensors demonstrate a maximum sensitivity of 0.088 kPa^−1^ for pressures below 10 kPa, increasing to 0.24 kPa^−1^ for pressures of 80 kPa. Furthermore, the developed sensors are successfully applied to measure heartbeats both before and after aerobic activity, showcasing their excellent sensitivity within the typical pressure range exerted by the heartbeat, which typically falls between 10 and 20 kPa.

## 1. Introduction

Recent advances in the field of wearable electronics and human–machine interface systems have sparked significant interest in developing flexible strain and pressure sensors. The key requirements for these sensors include flexibility, high sensitivity, and fast response times, enabling real-time monitoring of physical stimuli. In the last few years, new manufacturing techniques have contributed to the production of sensors based on highly engineered and functionalized materials [1,2].

The conversion of a mechanical stimulus, such as pressure or strain, into measurable electrical signals can be achieved by different transduction mechanisms, including: (i) The piezoelectric effect, based on the generation of charges; (ii) the piezo-capacitive effect, based on the capacitance variation; and (iii) the piezoresistive effect, based on the variation of the electrical resistance [3]. Piezoelectric pressure sensors are self-powered devices, but they are not able to measure static mechanical loads [4]. Capacitive sensors are suitable for both static and dynamic loads; however, the low capacitance variations (typically of the order of pF) and the need for a careful circuit to minimize parasitic effects make these devices difficult to use [5]. Due to their low cost, durability, robustness, and high resolution for both static and dynamic loads, piezoresistive sensors are highly suitable for applications such as wearable electronics, electronic skin, and similar fields [6]. These sensors indeed possess the capability to convert applied mechanical stress or deformation into electrical resistance variations. The behavior observed in composite materials, where the change in resistance is influenced by the formation or disruption of conductive paths due to the presence of electrically conducting fillers, is also observed in new porous elastomeric nanocomposite foams with open cells. These foams combine conductive nanomaterials such as graphene nanoplatelets (GNPs), carbon nanotubes (CNTs), and graphene with polymers such as polydimethylsiloxane (PDMS), Ecoflex^®^, or polyurethane (PU). Previous studies have shown that these materials exhibit both high piezoresistive properties and flexibility [3,7,8,9,10,11,12,13,14,15]. 

One of the most recent methods for producing piezoresistive sensors utilizing elastomeric nanocomposite foams is through direct 3D printing of customized polymeric filaments containing conductive nanoparticles. However, this type of process involves several issues. For instance, when employing high nanoparticle concentrations (>10 wt%), it can lead to a decline in the mechanical properties of the polymer [16]. Moreover, the direct printing of the conductive nanocomposite polymer can induce clogging of the 3D printer nozzle, posing difficulties for scaling up this technique. To address these challenges, researchers are exploring alternative approaches to utilize 3D printing in the field of sensors. Particularly interesting is the one that involves printing sacrificial templates, which are dissolved after polymer infiltration [17]. Unlike direct printing, this technique entails coating the fabricated samples with conductive nanoparticles (such as GNPs) only after the production of the elastomeric foam.

In our previous studies [13,14], we employed a sugar sacrificial template to create PDMS/Ecoflex^®^ foams coated with GNPs. These foams were utilized to produce medium- and low-pressure sensors with excellent sensitivity. However, one limitation was the presence of stochastic porosity in the polymeric foams. To regulate the porosity of the foams, in this work, we adopted the 3D printing technique to develop the cell geometry. We created an acrylonitrile butadiene styrene (ABS) sacrificial template, which served as the inverse replica of the foams. The design of the foams was accomplished using computer-aided design (CAD) software and subsequently printed using a commercial fused deposition modeling (FDM) 3D printer. Leveraging the capabilities of the 3D printer, we achieved greater control over the cell geometry of the sensors, thereby influencing their mechanical and electromechanical performance. In particular, we investigated two distinct structures for fabricating flexible open-cell foams: (i) Grids and (ii) triply periodic minimal surfaces (TPMS) [17]. The grid structure (GS) is a simple design that offers ease in adjusting the 3D geometry [18]. On the other hand, the TPMS-based structure (TPMSS) has gained significant attention within the scientific community due to its unique deformation mechanism [17,19]. Both structures were obtained using the sacrificial 3D printing technique, and our research aims to identify which structure provides greater flexibility, sensitivity, and control over the piezoresistive properties, especially in the low-pressure range.

As described in Section 2, the production of porous polymeric structures involves the simple leaching process of various ABS 3D templates that have been infiltrated with different commercial polymers. The selection of the appropriate polymer and filler was based on the optimization of mechanical properties, particularly aiming for the lowest Young’s modulus and the piezoresistive properties with the highest sensitivity. Several polymers were examined in the investigation, but ultimately, Solaris proved to be the most suitable for the development of our pressure sensors due to its superior mechanical properties and the necessary flexibility.

To achieve a piezoresistive foam, we infused the 3D porous structure with a colloidal suspension of GNP/1-propanol using drop casting. By integrating GNPs into the polymer structure, conductive paths were formed, leading to the inherent development of piezoresistive properties within the structure. Additionally, we elaborate on the determination of optimal printing parameters for fabricating the ABS sacrificial templates and the meticulous selection of the most suitable polymer for the infiltration process. These steps were essential in ensuring the successful implementation of the desired properties in the pressure sensors. Section 3 covers the comprehensive characterization of the produced samples, including various tests conducted to examine their properties. The morphology of the produced foams is investigated through field emission electron scanning microscopy (FE-SEM); then their mechanical behavior is assessed under quasi-static compression loads up to 90 kPa or 80 kPa, depending on the foams used (see Section 3.1.1), along with an assessment of their piezoresistive response after the infiltration process. The performance of pressure sensors is evaluated by considering their sensitivity and response stability to compression. Lastly, the foam material produced is integrated into a bracelet to monitor the heartbeat.

Our study revealed that the proposed sensors have a higher sensitivity compared to CNT-based solutions [20] and also with respect to our previous PDMS/GNP foams [13], reaching a maximum sensitivity of 0.088 kPa^−1^ for pressures below 10 kPa up to 0.24 kPa^−1^ for pressures of 80 kPa. Of particular interest to us is the sensitivity range between 10 and 20 kPa, as we aim to utilize our piezoresistive foams for measuring heart rate before and after aerobic activities, which commonly exert pressures within this range.

## 2. Materials and Methods

**Figure 1:**
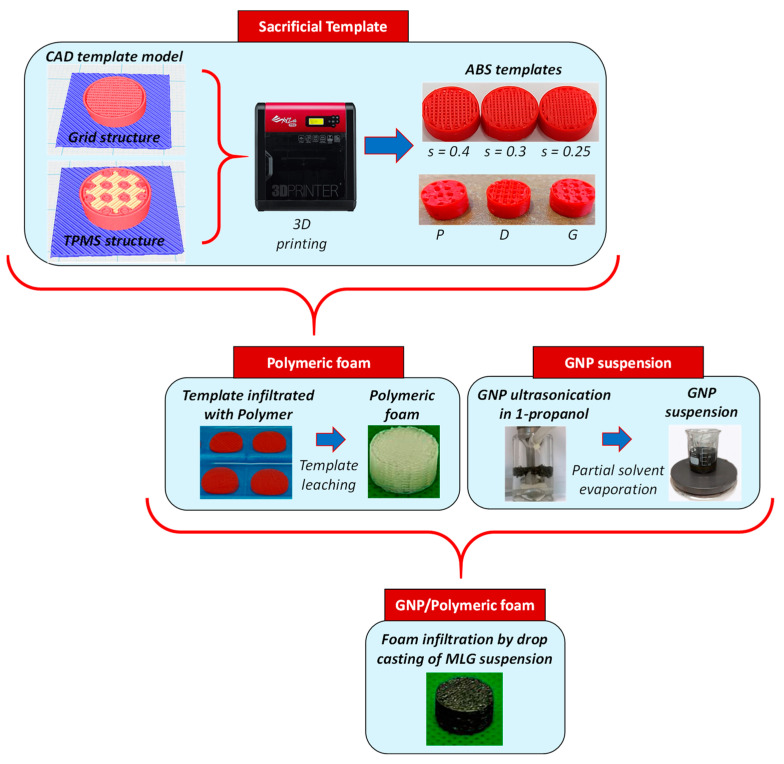
The schematic steps developed to fabricate the piezoresistive GNPs/polymer foams.

In this section, we describe the procedure developed to fabricate the soft, porous GNP/polymer foams that can be utilized as low/medium-pressure sensors. The production process, as depicted in Figure 1, involves five different steps: (i) 3D printing of ABS templates, which serve as a negative replica of the final samples; (ii) infiltration of the sacrificial template with low viscosity polymers; (iii) leaching of the ABS templates in acetone; (iv) preparation of GNP-based colloidal suspension; (v) realization of piezoresistive foams by infiltrating the open-cell lattice structure with the GNP colloidal suspension.

### 2.1. ABS Sacrificial Templates

In contrast to our previous works [13,14], where we obtained templates with stochastic-sized cells and limited control over porosity, in this paper we use a different approach. We produced ABS-structured templates designed with a CAD tool using an appropriate slicer software (XYZware Pro, V1.1.28.1), and these templates were then printed using a commercial FDM 3D printer (Da Vinci 1.0 Pro XYZ 3D printer, XYZprinting, Taipei, Taiwan), equipped with a 0.4 mm nozzle for extruding the ABS filament. The extrusion temperature is 210 °C, and the printing bed is maintained at 90 °C. Two types of geometries are investigated: (i) GS, inspired by the rectilinear filling of the 3D printer; (ii) TPMSS.

#### 2.1.1. Grid Structures (GS)

**Figure 2:**
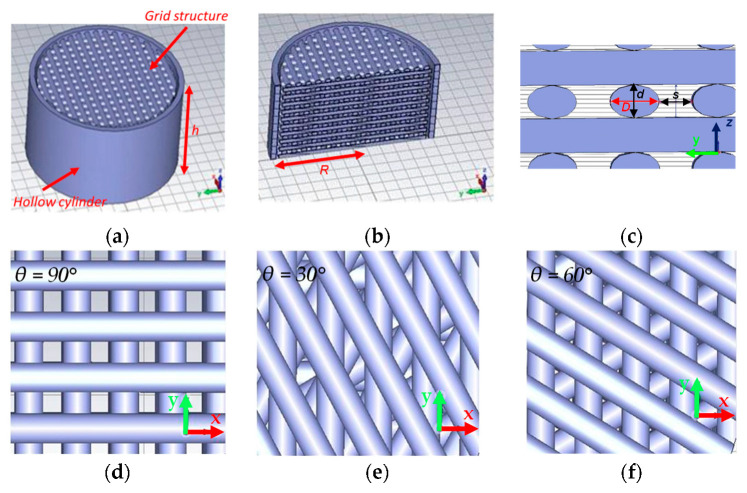
Model of a template with *θ =* 90° (**a**); section of the model in the yz plane (**b**); magnification of a section along the yz plane (**c**); magnification of a section along the xy plane for *θ* equal to 90°, 30 and 60° (**d**–**f**).

The GS of the sacrificial template consists of stacked layers, with each layer comprising spaced parallel cylinders that have elliptical cross-sections. The 3D model of the printed structure is depicted in Figure 2.

*D* and *d* are the major and minor axes of the ellipse, respectively (see Figure 2c). The former corresponds approximately to the extrusion width of the printer, whereas the latter represents the thickness of a single layer. Considering the adopted cartesian reference system, *d* is always directed along the *z* axis. The distance between two adjacent cylindrical segments is denoted as s. *D* and *d* are fixed at 0.43 mm and 0.30 mm, respectively. 

Two consecutive layers can be rotated relative to each other by an angle *θ*. In particular, Figure 2d–f shows a part of the section in the xy-plane of the structure in which *θ* is equal to 90°, 30°, and 60°, respectively. The GS has a total thickness of h=n·d, where *n* is the number of layers, inserted inside a hollow cylinder of the same thickness, *h* ~ 6 mm (Figure 2a), and an inner radius of *R* ~ 6 mm (Figure 2b).

Since *D* and *d* are fixed parameters, only the modification of *s* with the CAD tool allows control of the ratio of the volume of voids to the total volume of the ABS segments. Following the infiltration process, this ratio determines the porosity Φ of the final elastomeric foam, which serves as the negative replica of the template. Porosity (Φ) can be defined as:

(1) $$\Phi =\frac{V_{p}}{V_{t}}$$

where $V_{t}=\pi \cdot R^{2}\cdot h$ is the total (bulk) volume of the template and $V_{p}$ is the total pores’ volume, corresponding to the total volume of the ABS segments. The minimum value chosen for *s* is 0.2 or 0.25 depending on the type of infiltrated polymer used (as illustrated in Section 3.1), whereas the maximum value is 0.40 mm due to the limit of the 3D printer. The *θ* parameter has no influence on the porosity of the structure but strongly affects the softness of the foams, as will be discussed in the following paragraphs.

#### 2.1.2. TPMS Structures (TPMSS)

**Figure 3:**
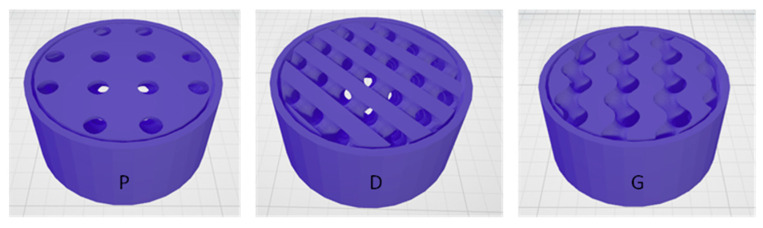
P, D, and G TPMS with external shell.

TPMSS are a type of implicit surface that exhibits periodicity in three independent Cartesian directions and has a mean curvature of zero [21]. Recently, these structures have gained attention for their applications in piezoresistive sensors [19]. In our study, we explored three distinct TPMS types: P, D, and G [17] (refer to Figure 3). In particular, based on their unit cells, we designed cylindrical sacrificial templates with the same total volume $V_{t}\;$as the GS.

### 2.2. Production of Polymeric Cell Structures

#### 2.2.1. Foam Production

The production of the open-cell foams involves modeling sacrificial templates made of ABS, followed by infiltration with a polymer. Before proceeding with the foaming process, we needed to investigate the effect of acetone on various non-porous cylindrical polymer samples (bulk structures). These bulk samples, with a thickness of 6 mm and a diameter of 12 mm, were produced using a brass mold and comprised seven different materials, including silicone rubbers and polyurethanes: PDMS (Dow, Midland, TX, USA), Ecoflex^®^ 00-20, Solaris, Mold Max 10T, Reoflex 20 Vytaflex 10, Dragon Skin 10 and Vytaflex 20 (Smooth-on, Macungie, PA, USA). 

To prepare the bulk samples, we thoroughly mixed the prepolymer (part A) and the curing agent (part B) for 5 min, adhering to the recommended ratio, which varies based on the specific polymer employed. After the mixing, we degassed the mixtures inside a desiccator at a pressure of ~313 mbar for 15 min to remove any entrapped air bubbles. Subsequently, the mixtures were poured into the brass mold and placed under a constant pressure of ~313 mbar at room temperature (RT = 24 °C) for 12 h. The curing process was completed by subjecting the samples to an oven at 150 °C for 10 min. After curing, the polymers were immersed in 80 mL of acetone and stirred at 400 rpm for 30 min. Among the tested polymers, Dragon Skin 10, Reoflex 20, Vytaflex 10, and Vytaflex 20 were damaged during the acetone bath, while PDMS, Solaris, Mold Max 10T, and Ecoflex^®^ 00-20 remained unaffected.

The foam fabrication process involved infiltrating the ABS templates with the selected polymers, as previously described in [9]. After curing, we dissolved the ABS templates in 80 mL of acetone with magnetic stirring at 400 rpm for 3 h. Finally, the resultant foams were placed in an oven at 100 °C for 5 min to ensure complete solvent evaporation. 

**Figure 4:**
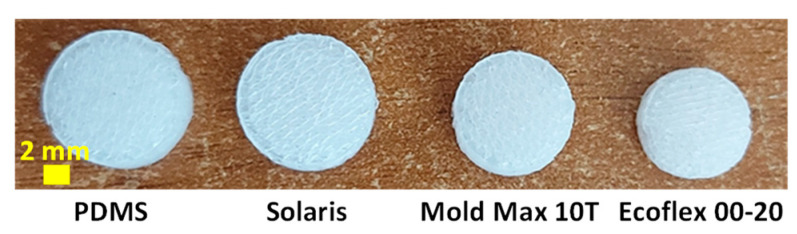
Acetone effect over the GS, produced with different polymers.

**Figure 5:**
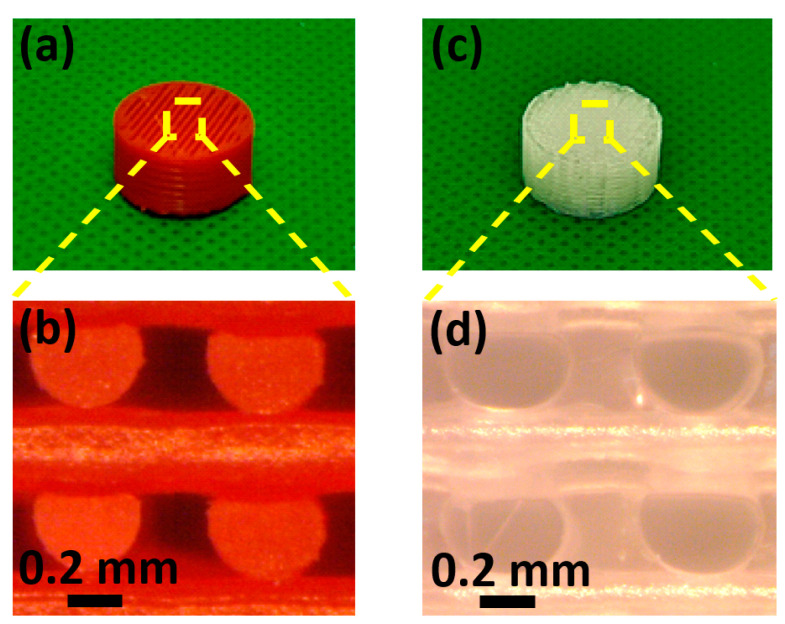
ABS template (**a**), optical microscope magnification of the template (**b**), corresponding cellular polymer structure (**c**), optical microscope magnification of the cellular structure (**d**).

The impact of acetone on the porous structures is significantly higher due to their much larger surface area compared to the bulk samples, as clearly depicted in Figure 4. We also assessed the effect of acetone on the foams by measuring their weight before and after the ABS template dissolution process, as shown in Table 1. Notably, PDMS and Solaris exhibited minimal weight reduction and size changes, indicating their robustness against the solvent. Consequently, we decided to focus our efforts on these two polymers for the fabrication of the piezoresistive foams. In Figure 5, we present an ABS grid template alongside its corresponding porous PDMS structure.

#### 2.2.2. GNPs Infiltration Process 

To create the piezoresistive porous cellular structures, we utilized a drop casting technique to infiltrate the foams with a colloidal suspension of graphene nanoplatelets (GNPs) in 1-propanol. The GNPs used in this process have an average thickness of 8–10 nm and lateral dimensions up to a few micrometers [13], making them suitable for the desired application. The infiltration process was based on our previous works [13,14] and involved the following steps:

- Dispersing the GNPs in 1-propanol with a concentration of 0.1 mg/mL and exfoliating them through ultrasonication using a sonotrode tip (Sonics & Materials Vibracell VC505, Sonics, Newtown, CT, USA) operating with a symmetric duty cycle (1 s on-phase, 1 s off-phase).
- Evaporating the solvent to obtain a final concentration of 1 mg/mL of GNPs.
- Infiltrating the polymeric structures with the GNPs/1-propanol solution using a 100 µL micropipette. To ensure a homogeneous distribution of GNPs, we alternated the faces of the sample every 60 s.

**Figure 6:**
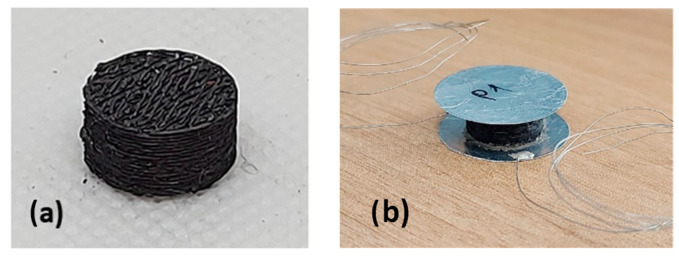
Cellular polymeric GS coated with GNPs (**a**), and with the conductive Al plates and wires (**b**).

For example, Figure 6 displays a PDMS grid porous structure that has been successfully infiltrated with GNPs. The foams underwent infiltration with varying amounts of the GNPs/1-propanol solution, specifically 0.6 mL, 1 mL, and 2 mL. After the infiltration process, the foams were sandwiched between two circular aluminum (Al) plates. To ensure better uniformity and connectivity with the Al electrodes, we used an epoxy-conducting resin to glue the plates. Subsequently, a conductive glue was employed to attach a wire to each electrode, serving as a connection for the subsequent electrical tests (see Figure 6b).

## 3. Results and Discussion

### 3.1. Mechanical Characterizations

**Figure 7:**
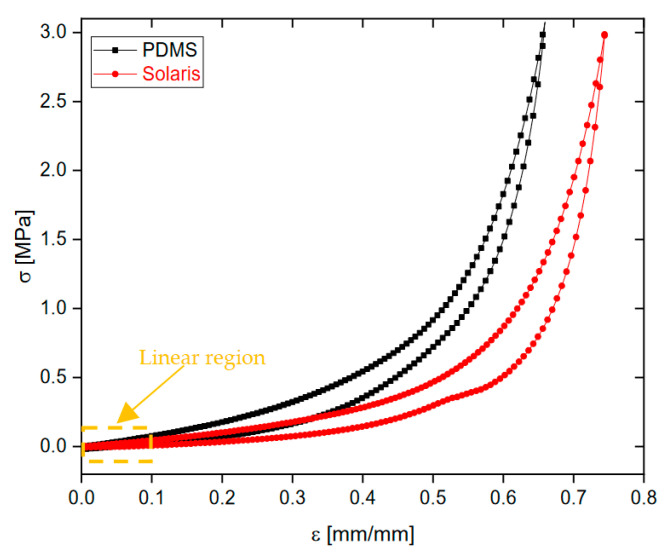
Comparison between the stress–strain curves of the bulk samples of PDMS and Solaris.

To determine the polymer with the best mechanical properties, particularly the lowest Young’s Modulus, stress–strain curves were performed on the bulk polymers using uniaxial compression mechanical tests. These tests were conducted on an Instron 3366 universal testing machine (Instron Worldwide Headquarters, Norwood (MA), USA). A 500 N load cell was used, and the maximum compressive stress applied was 3 MPa. The compression speed was set to 1 mm/min. Figure 7 illustrates the mechanical behavior of the bulk polymers, and Table 2 presents the corresponding Young’s modulus (E = d*σ*⁄d*ε* [MPa], where *σ* is the stress and *ε* is the strain). The Young’s modulus was evaluated from the initial slope of the stress–strain curve at 0.1 mm/mm deformation within the linear region. From the results, it is evident that Solaris exhibits a softer bulk and higher flexibility compared to PDMS, making it an excellent candidate for the development of flexible piezoresistive pressure sensors. This finding is crucial to the optimization of the sensor’s performance and sensitivity to mechanical stress or deformation.

Then the investigation focused on assessing the impact of printing geometric parameters on the mechanical behavior of PDMS polymer foams to identify the optimal values for achieving softness. Mechanical characterization tests were performed, applying a maximum compression stress of 90 kPa at a speed of 1 mm/min with a 10 N load cell.

**Figure 8:**
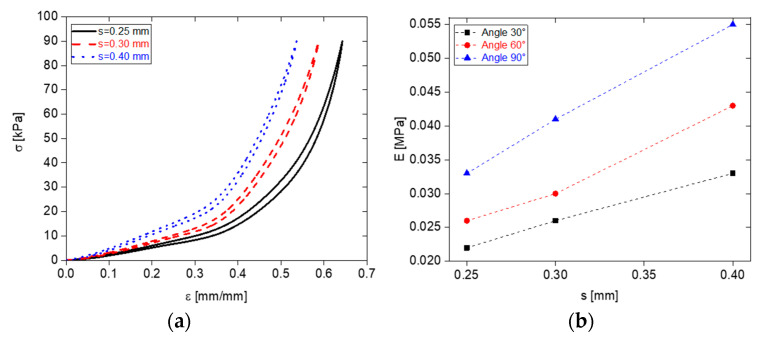
Stress–strain curves of PDMS foams for *θ =* 60° and different values of *s* (**a**); Young’s modulus as function of *s* and *θ* (**b**).

Initially, the angle *θ* was fixed at 60°, and the influence of the geometric parameter *s* was examined by varying its values (0.25 mm, 0.30 mm, and 0.40 mm). The average porosity values were observed to be 0.62, 0.59, and 0.53 for *s* values of 0.25 mm, 0.30 mm, and 0.40 mm, respectively. Figure 8a displays the results, indicating that decreasing the *s* value enhances the mechanical properties, specifically softness.

Additionally, the combined effect of the parameters *s* and *θ* was explored by considering different values of both. Young’s modulus was examined, and Figure 8b illustrates that the lowest modulus value, indicative of higher compressibility and increased softness, was achieved with *s* = 0.25 mm and *θ* = 30°. This finding emphasizes the ability to control the mechanical properties of the foams by manipulating the parameters *s* and *θ*. Reducing the values of both parameters leads to the production of softer foam samples.

**Figure 9:**
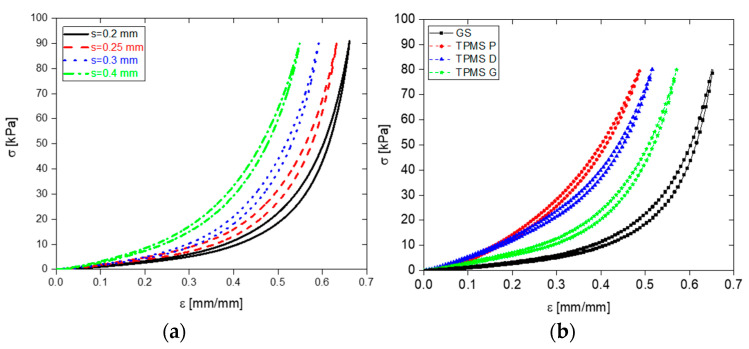
Comparison between the stress–strain curves of the Solaris foams based on the GS with *θ* = 30° and different values of *s* (**a**); comparison of stress–strain curves of the foams produced by TPMSS and optimized GS sacrificial templates (**b**).

Based on our findings, we made the decision to explore the use of the Solaris polymer, which allowed us to achieve a more precise value of *s* down to 0.2 mm. This level of precision was not achievable with PDMS, as it resulted in damage during the foam creation process. By utilizing Solaris with the reduced *s* value, we observed improved mechanical performance. Figure 9a illustrates the stress–strain curves of Solaris, highlighting the relationship between the *s* parameter and the mechanical behavior, with *θ* set to 30°. 

#### 3.1.1. Comparison with TPMSS

In the previous paragraph, we identified that soft foams can be fabricated using Solaris with an ABS grid template printed with the following parameters: *s* = 0.20 mm, *θ* = 30°. In this section, we compare the geometrically optimized GS to the foams fabricated using a sacrificial template based on TPMSS. 

The three types of TPMS (P, D, and G) were designed to maintain the same total volume as the GS. As can be observed from Figure 9b and the comparison of the Young’s modulus reported in Table 3, the optimized GS resulted significantly more flexible than the cellular structures obtained with TPMS-based geometry in all the investigated pressure ranges (0–80) kPa. This time, to ensure that the mechanical test configuration limits (10 N load cell) were not exceeded, the analysis was conducted within the range of 0 to 80 kPa. This range was chosen specifically considering the higher stiffness of the TPMSS.

### 3.2. Morphological Characterization

**Figure 10:**
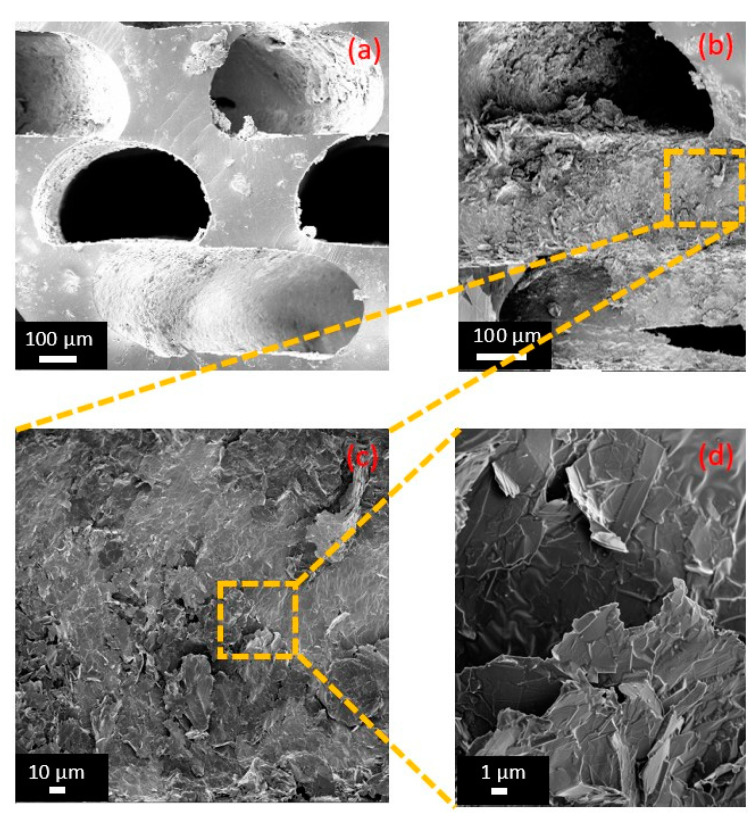
Cross-sections of porous GS with and without GNPs (**b**,**a**). Different magnifications of a cross-section of the polymer structure infiltrated with GNPs: (**c**) 1 kX and (**d**) 10 kX.

In order to analyze the morphology of the optimized GS, we utilized a Field-Emission Scanning Electron Microscopy (FE-SEM) system (Auriga, Carl Zeiss, Oberkochen, Germany) operating at a voltage of 2 kV. To prevent surface charging, all the samples were sputter coated with 20 nm of Cr using a Quorum Technologies Q150T ES sputter coater (Laughton, East Sussex, UK). Figure 10a,b shows the cross-sections of the GS without and with the GNPs. Figure 10c,d shows a magnification of the cross-sections of the GS infiltrated with the GNPs and highlights the presence of a network of nanoplatelets, which is responsible for the nanocomposite’s piezoresistive behavior. From the SEM images, we can estimate the average dimensions of the foam void channels to be approximately *D* ~ 450 μm, *d* ~ 300 μm, as expected by the CAD design.

### 3.3. Electrical Characterizations 

The electrical conductance (*G*_0_) of the foams under resting conditions was measured using a two-wire volt-amperometric technique employing a Keithley 6221 DC/AC current source and a Keithley 2182A nanovoltmeter (Keithley, Cleveland, OH, USA). The initial conductance value of the piezoresistive structures depended on the quantity of GNP colloidal suspension drop-cast. Three different amounts of GNP suspension were considered: 0.6 mL, 1 mL, and 2 mL. The structures infiltrated with 2 mL of solution exhibited the highest initial conductance, measuring approximately 1 mS, as there was a greater quantity of GNPs on the internal surfaces of the pores. For structures infiltrated with 1 mL of suspension, the conductance was lower, falling in the range of tens of μS. However, structures infiltrated with 0.6 mL of solution showed the lowest conductance in the range of μS. This value was considered too low for use in our pressure sensors, and therefore, we did not include it in the following characterizations. 

The average values of the conductance under resting conditions, calculated across all samples, are reported in Table 4.

### 3.4. Electromechanical Characterizations 

**Figure 11:**
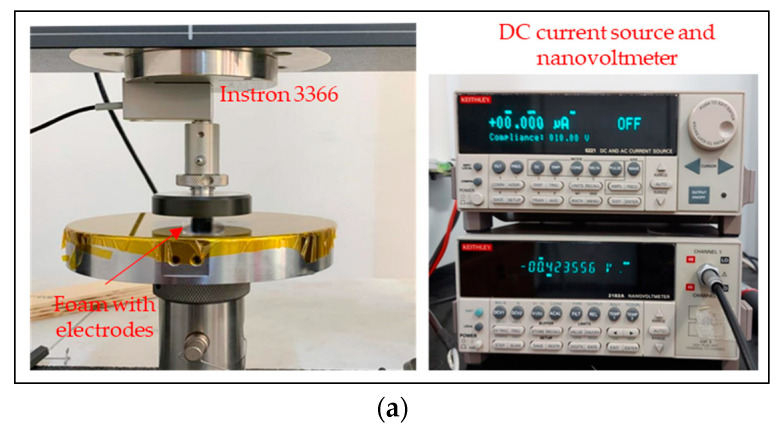

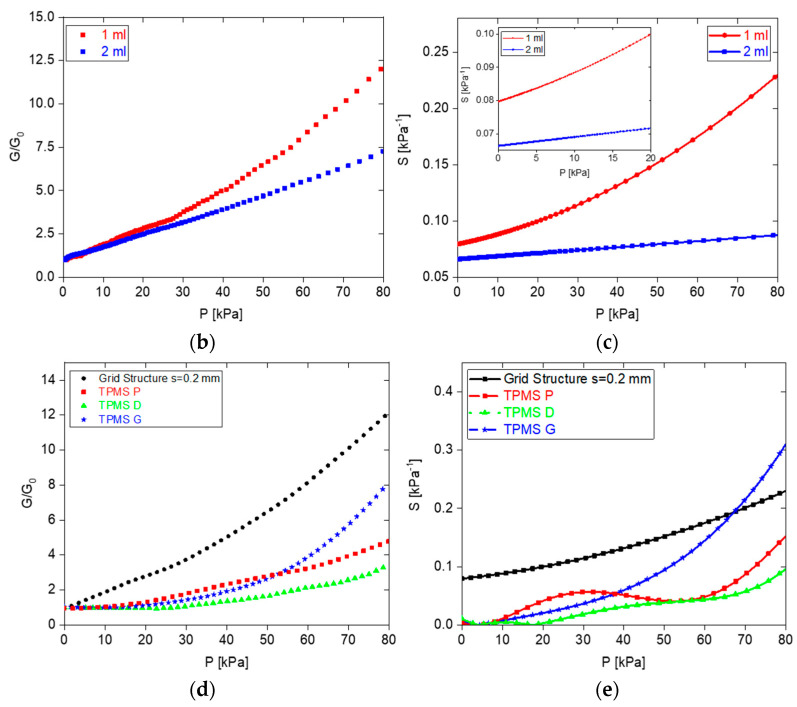
Image of the electromechanical setup (**a**); normalized conductance (**b**); sensitivity (**c**) of flexible piezoresistive sensors with 1 mL of suspension (red curve) and 2 mL of suspension (blue curve) as a function of pressure; normalized conductance (**d**) and sensitivity (**e**) of flexible piezoresistive sensors based on different cell structures as a function of the applied pressure.

To assess the piezoresistive behavior of the fabricated sensors, which were loaded with 2 mL and 1 mL of GNP suspension, we conducted multiple electromechanical characterizations, observing the variation of electrical conductance during various quasi-static uniaxial mechanical compression tests, including cyclic ones. The measurements were carried out using the previously described instrumentation (Figure 11a) at a constant temperature of approximately 21 °C and a constant relative humidity of approximately 43% RH. 

Specifically, we injected a DC current with an amplitude of 10 µA, and by measuring the voltage variation between the electrodes, we evaluated the change in conductance as a function of the applied load. In order to compare the electromechanical response of the infiltrated samples with 1 mL and 2 mL of the GNP colloidal suspension, we chose the optimized GS as a representative sample. Figure 11b shows the normalized conductance with respect to the applied stress. Depending on the amount of GNP suspension, we observed distinct behaviors in the foams. The normalized conductance exhibited a higher variation as the stress increased for the foams loaded with 1 mL of GNP suspension. 

To evaluate the performance of these two sample types more accurately, we calculated the sensitivity of the foams using the equation [14]:

(2) $$S=\left|\frac{dG}{dP}\right|\cdot \frac{1}{G_{0}}$$

where *S* is the sensitivity, *G* is the conductance, *G*_0_ is the initial conductance, and *P* is the applied pressure. The derivate was performed over the polynomial interpolated curves.

In Figure 11c, the sensitivities for pressure ranges of 0–80 kPa and 0–20 kPa, with 1 mL (red curve) and 2 mL (blue curve) of solution, are shown. It can be observed that the structures infiltrated with 2 mL of suspension have a sensitivity that remains practically constant as the applied pressure increases, stabilizing around a value of 0.07 kPa^−1^. This behavior can be explained by the presence of numerous initial conductive paths in the foams with 2 mL, resulting in a negligible, small variation in conductivity during mechanical compression. On the other hand, the samples with 1 mL of solution, which have fewer initial conductive paths, exhibit higher variation in conductivity and therefore greater sensitivity. At 80 kPa (medium pressure), the sensitivity reaches a value of 0.24 kPa^−1^, while at 10 kPa (low pressure), it reaches 0.088 kPa^−1^. Within the pressure range of 10–20 kPa, the sensitivity of the sensors ranges between 0.088 and 0.1 kPa^−1^.

Then, the electromechanical characteristics of the GS were compared to those of the TPMSS. In all cases, a 1 mL solution was utilized since, as observed in the optimized GS, it yields the highest sensitivity. The results in Figure 11d demonstrate that the GS exhibits a greater variation in conductance compared to the TPMSS as the uniaxial compressive stress increases. Specifically, the conductance of the TPMSS remains relatively flat up to a pressure of 15 kPa.

This characteristic is particularly pronounced in the D structure, which exhibits insensitivity until a pressure of 20 kPa is reached (Figure 11e). From our observations, we can conclude that up to *P* ≈ 67 kPa, the GS displays higher sensitivity compared to the TPMSS. This can be attributed to the smaller pore size and increased mechanical flexibility of the GS.

Hence, the GS with optimized geometrical parameters exhibits the greatest potential for manufacturing low-pressure sensors (*P* < 10 kPa) and heart rate sensors (10 kPa < *P* < 20 kPa), which is our range of interest. In Table 5, we summarize the performance parameters of different pressure sensors.

**Figure 12:**
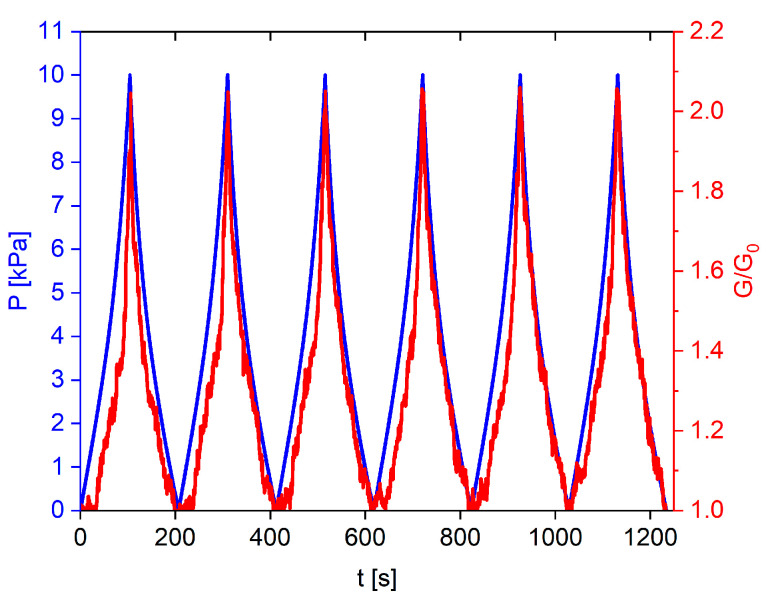
Conductance variation of GS (red) during six consecutive cyclic compression tests in the pressure range 0–10 kPa (blue).

Finally, in order to assess the stability of GS sensors, we performed cyclic loading/unloading compression tests within the low-pressure range of 0–10 kPa. The results depicted in Figure 12 illustrate the normalized conductance over time and pressure, showcasing a consistent and repeatable response.

### 3.5. Development of Heart Rate Sensors

To test the practical application of the sensors developed in this study, we fabricated a bracelet for heart rate detection. Heart rate can be manually measured by directly examining the pulse from the wrist or through auscultation. An electrocardiogram (ECG) is commonly used for detecting abnormal heart contractions. While ECG provides detailed information about the patient’s cardiac system, the equipment required for this type of analysis is complex and not easily replicable for home and sports applications. Most commercially available wearable devices designed for monitoring daily activities rely on optical techniques such as photoplethysmography, commonly integrated into smartwatches [22]. However, the inflexibility of the photoelectric modules, which necessitate direct skin contact, imposes limitations on their comfort, particularly during extended periods of use.

**Figure 13:**
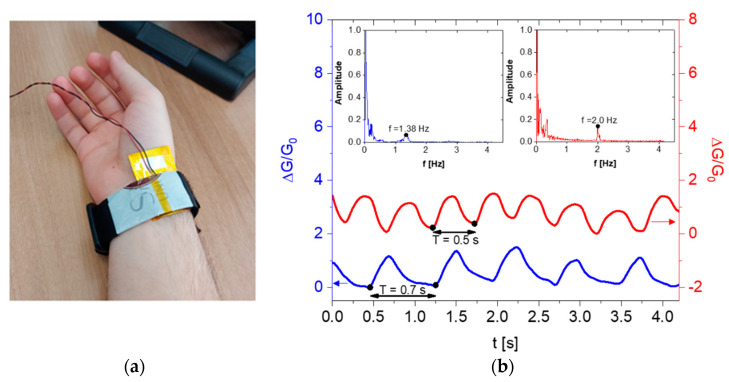
Bracelet with flexible piezoresistive sensor for heart rate detection (**a**); pulse signal at rest (blue) and after 5 min of aerobic activity (red) over an interval of 4 s (**b**).

On the other hand, flexible pressure sensors that are in direct contact with the skin offer the potential to detect arterial frequency by measuring variations in pressure within the wrist blood vessel. To achieve this, we utilized a bracelet that incorporates our best piezoresistive GS foam material. To reduce the distance between the skin and the sensor, as well as the stiffness of the device, two thin aluminum papers were used as electrodes instead of aluminum plates (see Figure 13a).

The electrical measurements were conducted using the same instrumentation employed for the electromechanical characterizations described in the previous paragraph. The bracelet was worn on the user’s wrist to measure the corresponding conductance at rest and after 5 min of aerobic activity. Figure 13b displays the normalized conductance variation as a function of time before and after the activity. At rest, the signal’s local maxima are approximately 0.7 s apart, indicating a heart rate of approximately 80 beats per minute (bpm). However, after 5 min of aerobic activity, the interval between the peaks decreases to around 0.5 s, corresponding to a heart rate of approximately 120 bpm. These findings are further supported by the fast Fourier transform (FFT) analysis of the signals, as shown in the insets of Figure 13b.

## 4. Conclusions

In this work, highly flexible piezoresistive pressure sensors have been developed using sacrificial 3D-printed templates. The ABS sacrificial templates were 3D printed using a low-cost technology based on the FDM method and then dissolved with acetone to obtain the polymeric open-cell structures. Different types of cellular polymeric structures were investigated, including grid structures controlled by different parameter values (*s* and *θ*), as well as TPMS of types P, D, and G.

The mechanical tests conducted provided insights into the influence of design parameters of 3D-printed sacrificial templates on the flexibility and electromechanical properties of the resulting foams. Among these parameters, the GS exhibited superior flexibility compared to the TPMSS. To fabricate the foams, Solaris was chosen as an elastomer due to its remarkable flexibility and resistance to acetone. Through experimental tests, we determined the optimal geometric parameters for the GS, prioritizing softness, to be *s* = 0.20 mm and *θ* = 30°. As mentioned in Section 3.3, the electromechanical response of the sensors is influenced by the quantity of infiltrated GNP solution. The produced sensors exhibited a positive piezoresistive effect as the applied pressure increased during the uniaxial compression tests. Using 2 mL of GNP solution resulted in excessively high conductance (*G*_0_ ~ 1 mS), with a nearly constant sensitivity of 0.07 kPa^−1^. On the other hand, with 0.6 mL, *G*_0_ was too low (<1 µS) for practical use, inhibiting an accurate evaluation of sensitivity. Therefore, all samples were infiltrated with 1 mL of solution, resulting in a *G*_0_ value of approximately 74 µS and a sharper change in sensitivity, with the highest values observed as *S* = 0.088 kPa^−1^ for *P* = 10 kPa and *S* = 0.24 kPa^−1^ for *P* = 80 kPa.

When comparing the optimized GS with different types of TPMSS, we observed a more pronounced piezoresistive effect in the former. On the other hand, the TPMS structures exhibited no response until a pressure of 15 kPa was reached, displaying nearly zero sensitivity. As a result, in the low-pressure range, the foams with GS were the only ones capable of providing high sensitivity. Furthermore, as evidenced by the cycle tests, these foams exhibited reliable response repeatability.

Within the pressure range of 10 kPa to 80 kPa, we noticed that the GS maintained a higher sensitivity compared to TPMSS, except for the G-type structure, where its sensitivity surpassed the GS’s one above 67 kPa. 

The GS demonstrated excellent characteristics for fabricating low- to medium-pressure (especially low-pressure) sensors suitable for various applications. Notably, we successfully demonstrated its application as a heart rate sensor.

## Figures and Tables

**Table 1:** Variation of the weight before and after the acetone bath.

Polymer	Initial Weight [g]	Final Weight [g]	Δ [g]
Solaris	0.242	0.208	0.034
Mold Max 10T	0.285	0.170	0.115
Ecoflex^®^ 00-20	0.250	0.126	0.124
PDMS	0.255	0.222	0.033

**Table 2:** Young’s modulus of the produced bulk samples.

Polymer	E [MPa]
Solaris	0.369
PDMS	0.699

**Table 3:** Young’s modulus of the produced foam samples.

Structure	E [kPa]
Grid	16
TPMS P	68
TPMS D	62
TPMS G	35

**Table 4:** Conductance value at rest condition of the foams loaded with different concentrations of GNPs.

GNPs Suspension [mL]	G_0_
2	(1.00 ± 0.38) mS
1	(73.81 ± 8.58) μS
0.6	<1 μS

**Table 5:** Performance parameters of different pressure sensors.

Structure	E [kPa]	G_0_ [μS]	S [kPa^−1^] @ 10 kPa	S [kPa^−1^] @ 80 kPa
Grid	16	73.81	0.088	0.24
TPMS P	68	52.49	0.011	0.15
TPMS D	62	97.91	0.005	0.10
TPMS G	35	67.07	0.008	0.31

## Data Availability

Not applicable.
